# A Case of Intermittent from Birth, and Its Subsequent Progress

**Published:** 1843-08

**Authors:** B. W. Avent

**Affiliations:** Murfreesborough, Tennessee


Art. V.—*A Case of Intermittent from Birth, and its subsequent progress.*—Reported to the Medical Society of Tennessee. By Dr. B. W. Avent, of Murfreesborough, Tennessee.

During the early part of the summer of 1839, a lady in her fifth month of pregnancy, who lived near one of the water courses in the neighborhood of Murfreesborough, was attacked with intermittent fever, assuming a tertian form. She took the usual remedies advised in cases of this character, and in a short time the chills left her. In two weeks, however, she had a return of them, and was again relieved by similar remedies. But it seemed that the cure still failed to be permanent, and she soon became the subject of a chronic form of intermittent. Owing to some prejudices, growing out of her condition, she did not again apply for medical aid, but up to the time of her delivery was considered in bad health, looked pale, and had dropsical swellings—every change of weather, or variation from the strictest course of regimen, produced a recurrence of the disease.

On the morning of the 21st September, she had a chill, as had been the case on every other day, for the past two weeks. On the going off of the fever, which followed the chill to day, she complained of uterine pains, which continued to in crease until evening, when I was requested to visit her. A natural though tedious labor was completed at nine o'clock on the following morning. The child was of common size, and breathed and cried healthily. I visited my patient, or rather patients, again in the evening, and learned that the child had been suffering very much (in the language of the nurses and friends) from cold—that its extremities had become cold and shrivelled, its lips blue, and its breathing disturbed; so alarming indeed were the symptoms, that they believed the little sufferer to be dying. This cold stage, though, had now given way to a general and pretty free re-action throughout the system. I saw no alarming symptoms in the case, and satisfied the family that such a state of things as they had mentiond, was not of unfrequent occurrence, and that, at present, there seemed to be nothing calculated to excite alarm.

On the following morning, I found the child seemingly well—but as it was the day in course for the mother's chill, I soon discovered that she would not escape. I now put her under treatment, and with some difficulty finally succeeded—by the end of the *month* in relieving her entirely. She had no more chills after she got up. But to recur to the child—during this day, it continued to do well, sucked heartily, and slept on as young children usually do.

On the next day, 24th of September, I called during the morning, and satisfied myself as to the improvement of the mother's condition, and having only enquired after the child and learning that it was well, I did not examine its situation. But in the evening it was not so well—and at the same hour that the alarming symptoms appeared on the first day of its birth, it was again attacked, in the same way—*yawned, stretched, grew cold, lips became blue, and breathing again interrupted*—these symptoms lasted an hour, and gradually subsided, and I have no doubt from the report of the family, were followed by fever.

On the 25th, I visited the family, when I learned the history of the preceding day, as I have related. The child had no fever this morning, but was evidently pale, feeble, and fretful; I gave it no medicine and promised to return on the morrow.

Twenty-sixth: I learned this morning that the child had no return of the unpleasant symptoms during the day before. I concluded to remain this day, and watch the case; I gave no medicine with a view to producing a change in its condition, and suspecting that this was becoming a regular case of intermittent fever, waited patiently, and watched closely every symptom. In the evening there was a recurrence of the cold stage, at the same hour of the previous days, and all the leading symptoms of confirmed intermittent were present. Fever followed and lasted some two hours or more, and was carried off by profuse perspiration, so much so, that I was alarmed for the safety of my little patient. Concluding that a treatment was now necessary, and it being a case of *ague and fever* I determined to use ague and fever medicine. I gave it half a grain of calomel, which operated well on its bowels during the night.

Twenty-seventh: on this day quinine was used, but as it was not the day for the chill, I used it in small doses and at long intervals. On the 28th the quinine was used more freely and kept up regularly, until past the time for the chill to come on. The patient this evening escaped, and I was satisfied the medicine had done well. I now advised the family to use the quinine in small doses on the expected days for the chill, so as entirely to break up if possible the predisposition to the disease. I heard nothing more from the case until October 1st, five days afterwards, when I learned that the child was again attacked, and that the symptoms were similar to the previous attacks. The quinine was again used with success; after this the child continued to improve slowly, and grew some, but still looked pale, and was thought not to be well.

About the 1st of November, the disease returned, assuming the same character, and was temporarily relieved by the same remedies. I now found that this disease had become completely fixed upon my little patient, and that other remedies besides quinine must be resorted to, or a cure could not be affected. I tried some of the preparations of iron, and in a word all the various *specifics* and *nostrums* for the cure of intermittents were successfully used, but had no other effect than that of affording temporary relief.

For twenty months the disease continued to attack the patient, and up to the present time, a period of more than three years, this child is still weakly, pale and small—though at present he is in better health than he has ever been, and has not had a chill for about four months. Hopes of his entire recovery are now entertained. I cannot say what remedy did most good, or most harm in this case; suffice it to say, that nothing afforded more than temporary relief.

An argument in favor of three very important principles in pathology, I think, may be adduced from this case: First. that *intermittent fever* may be congenital. Second. That the great cause, whatever it may be, which produces this affection, conveys its poison through the circulating system. Thirdly. That intermittent fever is a disease *sui generis.*

